# Iodine avidity in papillary and poorly differentiated thyroid cancer is predicted by immunohistochemical and molecular work-up

**DOI:** 10.1530/ETJ-23-0099

**Published:** 2023-07-28

**Authors:** Joachim N Nilsson, Jonathan Siikanen, Vincenzo Condello, Kenbugul Jatta, Ravi Saini, Christel Hedman, Catharina Ihre Lundgren, C Christofer Juhlin

**Affiliations:** 1 Department of Molecular Medicine and Surgery, Karolinska Institutet, Stockholm, Sweden; 2 Department of Medical Radiation Physics and Nuclear Medicine, Karolinska University Hospital, Stockholm, Sweden; 3 Department of Oncology-Pathology, Karolinska Institutet, Stockholm, Sweden; 4 Department of Pathology and Cancer Diagnostics, Karolinska University Hospital, Stockholm, Sweden; 5 Stockholms Sjukhem Foundation's Research and Development Department, Stockholm, Sweden; 6 Department of Clinical Sciences Lund, Lund University, Lund, Sweden; 7 Department of Breast, Endocrine Tumours and Sarcoma, Karolinska University Hospital, Stockholm, Sweden

**Keywords:** thyroid cancer, iodine avidity, TERT, NIS, TPO

## Abstract

**Background:**

Successful radioiodine treatment of differentiated thyroid cancer requires iodine avidity: that is, the concentration and retention of iodine in cancer tissue. Several parameters have previously been linked with lower iodine avidity. However, a comprehensive analysis of which factors best predict iodine avidity status, and the magnitude of their impact, is lacking.

**Methods:**

Quantitative measurements of iodine avidity in surgical specimens (primary tumour and lymph node metastases) of 28 patients were compared to immunohistochemical expression of the thyroid-stimulating hormone receptor, thyroid peroxidase (TPO), pendrin, sodium–iodide symporter (NIS) and mutational status of *BRAF* and the *TERT* promoter. Regression analysis was used to identify independent predictors of poor iodine avidity.

**Results:**

Mutations in* BRAF* and the *TERT* promoter were significantly associated with lower iodine avidity for lymph node metastases (18-fold and 10-fold, respectively). Membranous NIS localisation was found only in two cases but was significantly associated with high iodine avidity. TPO expression was significantly correlated with iodine avidity (*r* = 0.44). The multivariable modelling showed that tumour tissue localisation (primary tumour or lymph node metastasis), histological subtype, TPO and NIS expression and TERT promoter mutation were each independent predictors of iodine avidity that could explain 68% of the observed variation of iodine avidity.

**Conclusions:**

A model based on histological subtype, TPO and NIS expression and TERT promoter mutation, all evaluated on initial surgical material, can predict iodine avidity in thyroid cancer tissue ahead of treatment. This could inform early adaptation with respect to expected treatment effect.

## Introduction

Differentiated thyroid cancer is treated with surgery, and in cases of larger tumours or cervical lymph node metastases, additional hormone suppression and radioiodine therapy are given. Successful radioiodine therapy requires adequate uptake and trapping of iodine in the target tissue, which is conceptualised as iodine avidity. For most patients with low-risk disease, post-surgical treatment includes thyroid remnant ablation – a highly iodine avid target. However, in the treatment of patients with known metastases, or at high risk of developing such metastases, the main target consists of distant or lymph node metastases of unknown avidity. Due to iodine avidity being unknown, it is currently the size of the primary tumour and the presence of any initial lymph node metastases that mainly guide the choice of radioiodine treatment activity (1).

Several factors have been associated with lower iodine avidity of metastatic tissue, such as high patient age, large tumour size, histological type (follicular or papillary) and high [^18^F]fluorodeoxyglucose uptake (2, 3, 4, 5). Furthermore, tumours exhibiting *BRAF V600E* or *TERT* promoter mutations are less likely to spawn iodine avid metastases and are associated with poorer patient outcomes (6, 7, 8, 9). The co-occurrence of these two mutational events in papillary thyroid cancer (PTC) has been found to be especially indicative of aggressive tumour features (10).

The *SLC5A5* gene encodes the sodium–iodide symporter (NIS) protein, which is central in the transport of iodine into thyroid cells, and its frequently decreased expression and function in thyroid cancer limits the effectiveness of radioiodine (11). Loss-of-function mutations in NIS are very rare in thyroid cancer, and the reduced iodine transport exhibited by most thyroid cancer cells appears to be related to silencing or post-translational changes prohibiting the essential plasma membrane localisation of NIS (12, 13, 14). Several other proteins also have a large impact on the regulation and machinery of cellular iodine transport, such as thyroid peroxidase (TPO), thyroid-stimulating hormone receptor (TSHR) and pendrin (*SLC26A4*) (15).

However, all mentioned works linking various factors to iodine avidity have been based on post-therapeutic scintigraphic images. The classification of iodine avidity in previous research has been based on regional and distant metastases, often as a binary parameter (uptake yes/no), which limits the ability to accurately quantify the uptake.

This study aimed to provide quantitative information on iodine avidity with higher precision and detail than previously published, using *ex vivo* measurements of iodine concentrations in fresh tumour tissue from surgery. This enabled a unique evaluation of the iodine avidity in primary tumour tissue, resected lymph node metastases, and comparison of avidity to the expression of iodine-transport related proteins (NIS, TPO, TSHR and pendrin), as well as mutations in the *TERT* promoter and codon V600 of the *BRAF* gene.

## Materials and methods

### Patient selection

Patients referred to the Karolinska University Hospital, Stockholm, Sweden, with cytologically confirmed PTC estimated as larger than 1 cm by ultrasound (the general threshold for radioiodine treatment at our institution), were informed and queried for study participation. All adult patients that could understand the study information were considered for participation. The study has been approved by the Swedish Ethical Review Authority (#2020-01222 and #2020-01541), and all subjects signed an informed consent form prior to inclusion. Exclusion criteria were pregnancy and severe renal impairment (eGFR < 30 mL/min/1.7 m^2^). The patients were subsequently excluded if the primary tumour was too small and specimen collection at grossing, therefore, would risk compromising the histopathological diagnosis. Tissue specimens from 28 patients, collected between 2019 and 2021, were analysed. The data included primary tumour samples of PTC (21 patients), of differentiated high-grade thyroid cancer (DHGTC; 1 patient), of poorly differentiated thyroid cancer (PDTC; 3 patients) and lymph node metastases of PTC (11 patients), all removed at initial surgery. In 8 of the 11 patients, data on primary tumour and synchronous lymph node metastases was available; in the remaining 3 patients, no data could be collected from the primary tumour due to small lesions or unclear localisation.

### Histological analysis

Histopathological subtyping was performed in accordance with the 2022 World Health Organization classification. Notably, it includes a classification for the novel entity DHGTC: tumours with differentiated growth patterns and/or PTC-associated nuclear changes but with necrosis or an elevated number of mitoses (16).

### Sample and radioactivity handling

The methodology of sample and radioactivity handling has been described in detail in a previous publication, studying other aspects of iodine avidity in a part of this cohort (17). Here follows a shorter summary, with more details in Supplementary Material 1 (see section on supplementary materials given at the end of this article). Two days prior to surgery, the patients received a low-activity tracer injection of iodine-131 (5-10 MBq). The low activity and short time period between injection and surgery ensured that absorbed doses to tumour tissue were far below (<0.1 Gy) what has previously been described to impact iodine uptake or *NIS* mRNA expression (18), see Supplementary Material 2 for details. After surgery, representative pieces of tumour and lymph node metastases were dissected by an experienced surgical pathologist or a specialised pathology laboratory assistant. In the case of multifocal primary tumour growth, the largest lesion was dissected. The radioactivity in tumour samples was quantified as normalised activity concentration (fraction of injected activity per gram of tissue: IA g^−1^) by measurements in a gamma-counting scintillator detector. After radioactivity measurements were concluded, samples were fixed in formalin and embedded in paraffin (FFPE).

### Molecular pathology

Digital droplet polymerase chain reaction (ddPCR) analysis was used to screen for *TERT* promoter mutations (both C228T and C250T), as ddPCR has shown superior sensitivity compared to Sanger sequencing (19). Positive mutation calling was noted if the fractional abundance exceeded 3%. The mutational status of the *BRAF* gene (exon 15) was analysed by direct sequencing according to standard procedures. *BRAF* mutation-positive cases were also interrogated for point mutations in the RAS gene family by examining codons 12, 13 and 61 for *H-N-KRAS* in order to exclude any competing driver mutations. More details of molecular pathology methods are described in Supplementary Material 1.

### Immunohistochemical staining

FFPE material from all thyroid samples was used for immunohistochemical analysis of NIS, TPO, TSHR, pendrin and, as a complement to PCR analysis, B-Raf V600E protein expression. The staining of each marker was assessed by an endocrine pathologist (C C J). The level of immunoreactivity and sub-cellular localisation was evaluated and scored on a scale of negative (<10% of cells showing expression), weakly positive (25% of cells showing expression), moderately positive (50% of cells showing expression), strongly positive (75% of cells showing expression) and totally positive (100% of cells showing expression). More details of immunohistochemical staining methods can be found in Supplementary Material 1. Positive controls (Graves’ disease), negative controls (without primary antibody) and plasma membrane-specific stains performed for subsets of cases are shown in Supplementary Material 3.

### Statistical analysis

All analyses were performed using R (version 4.2.2, R-project.org). Data points on iodine avidity were log-transformed before statistical testing since their distribution was found to be approximately log-normal. For patients where multiple samples were taken from either primary tumour or lymph node metastases, the geometric mean of samples was calculated and used in any further analysis; only the resulting mean value for either primary tumour or lymph node metastases was used in the analysis and shown in the Results section. For tests between dichotomous groups, Welch’–s *t*-test was used, since sample variance was not evidently equal between groups. Associations between continuous variables were estimated using Pearson’s product–moment correlation. Multivariable regression was performed using a linear regression model (*lm* function in R). The model was optimised with respect to maximising adjusted *R* ^2^ while keeping bias and number of variables as low as possible. The model parameters were chosen after an initial stepwise algorithm including all variables was used to decrease collinearity and number of variables, with the intention to limit any overfitting. The normality of residuals was evaluated with quantile–quantile plots. Multicollinearity in the model was evaluated using variance inflation factors (*VIF* function in R). Skedasticity was evaluated using the Goldfeld-Quandt test (*gqtest* function in R). Power calculations were performed with limited data on the variance in iodine avidity for the different subgroups; a s.d. of 2.5E-5 IA g^−1^ and effect size of 5E-5 IA g^−1^ was used. A sample size of 25 was predicted to detect a two-fold difference in iodine avidity with 90% power in the rarest mutation (10% occurrence). In the ‘Results’ section, two-sided 95% confidence intervals (CIs) are reported throughout.

## Results

### Links between iodine avidity, immunohistochemical expression and mutations

**Figure 1:**
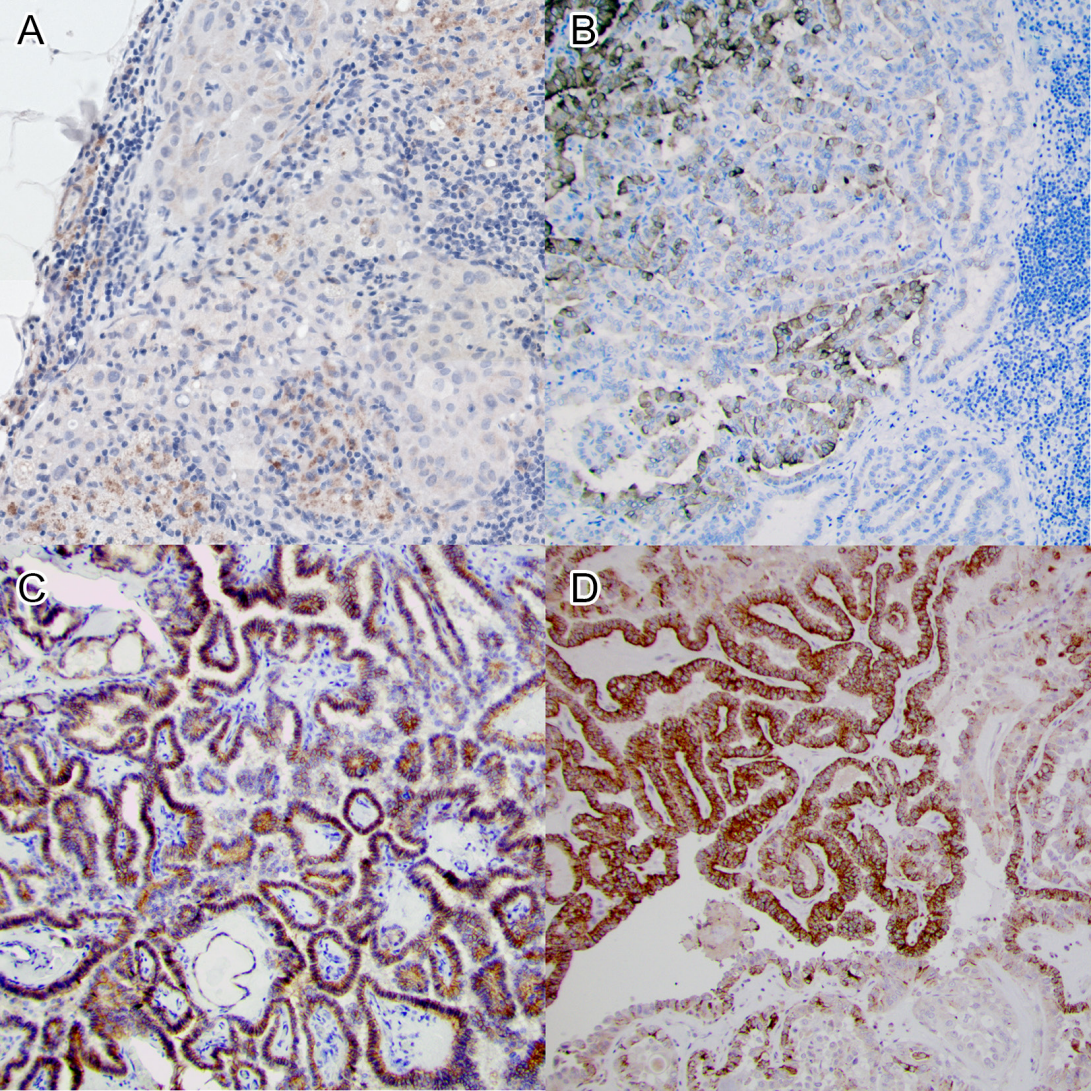
Examples of immunohistochemistry for the studied iodine-transport-related markers with haematoxylin (blue) as a counterstain in all images. NIS expression (brown) in a sample of lymph node metastases is shown in (A). The sample was scored as 50% of cells expressing NIS. Expression of TPO (brown), scored as 50%, in lymph node metastasis is shown in (B). TSHR expression (brown) was scored as 100% in a primary tumour sample in (C). Finally, pendrin (brown) was expressed in 75% of cells in a primary tumour sample shown in (D). The stroma present in the samples themselves served as negative controls for immunoreactivity. NIS, sodium–iodide symporter; TPO, thyroid peroxidase; TSHR, thyroid-stimulating hormone receptor.

Patient characteristics, the frequency of mutations in *BRAF* V600E and *TERT* C228T promoter mutations, and expression of NIS, TPO, TSHR and pendrin are shown in Tables 1, 2 and 3. Examples of immunohistochemical expression results for each antibody are shown in Fig. 1. Mutational analysis and NIS staining failed in one primary tumour sample, TSHR and pendrin staining was inconclusive in one primary tumour sample each, and TPO was inconclusive in one primary tumour and in one lymph node metastasis sample. These samples are therefore not reported in Tables 1, 2 and 3.

**Table 1:** Patient characteristics and histological subtypes.

Parameter	Total (*n* = 28)
Age	
Median (range)	53 years (19, 81)
Sex	
Male/female (proportion)	11/17 (39/61%)
Histological subtype	
Classic PTC	9 (32% of patients)
Diffuse sclerosing PTC	4 (14% of patients)
Warthin-like PTC	2 (7% of patients)
Follicular variant PTC	1 (4% of patients)
Oxyphilic PTC	1 (4% of patients)
Tall cell PTC	7 (25% of patients)
Differentiated high-grade thyroid cancer (DHGTC)	1 (4% of patients)
Poorly differentiated thyroid cancer (PDTC)	3 (11% of patients)

**Table 2:** Frequencies of mutations.

	*BRAF* V600E mutation	*TERT* promoter mutation (C228T)	*BRAF* and *TERT* mutation
Primary tumours	75% (18/24 patients)	33% (8/24 patients)	33% (8/24 patients)
Lymph node metastases	55% (6/11 patients)	36% (4/11 patients)	36% (4/11 patients)

**Table 3:** Immunohistochemical expression.

	Cytosolic NIS expression (≥50% of cells)	Membranous NIS expression (any % of cells)	TPO expression (≥50% of cells)	TSHR expression (≥50% of cells)	Pendrin expression (≥50% of cells)
Primary tumours	42%	8%	42%	88%	92%
	(10/24 patients)	(2/24 patients)	(10/24 patients)	(21/24 patients)	(22/24 patients)
Lymph node metastases	64%	0%	50%	64%	100%
	(7/11 patients)	(0/11 patients)	(5/10 patients)	(7/11 patients)	(11/11 patients)

Immunohistochemical results are presented as number of patients with more than half of cells (≥50%) expressing the respective protein, except for membranous NIS.

TPO, thyroid peroxidase; TSHR, thyroid-stimulating hormone receptor.

**Figure 2:**
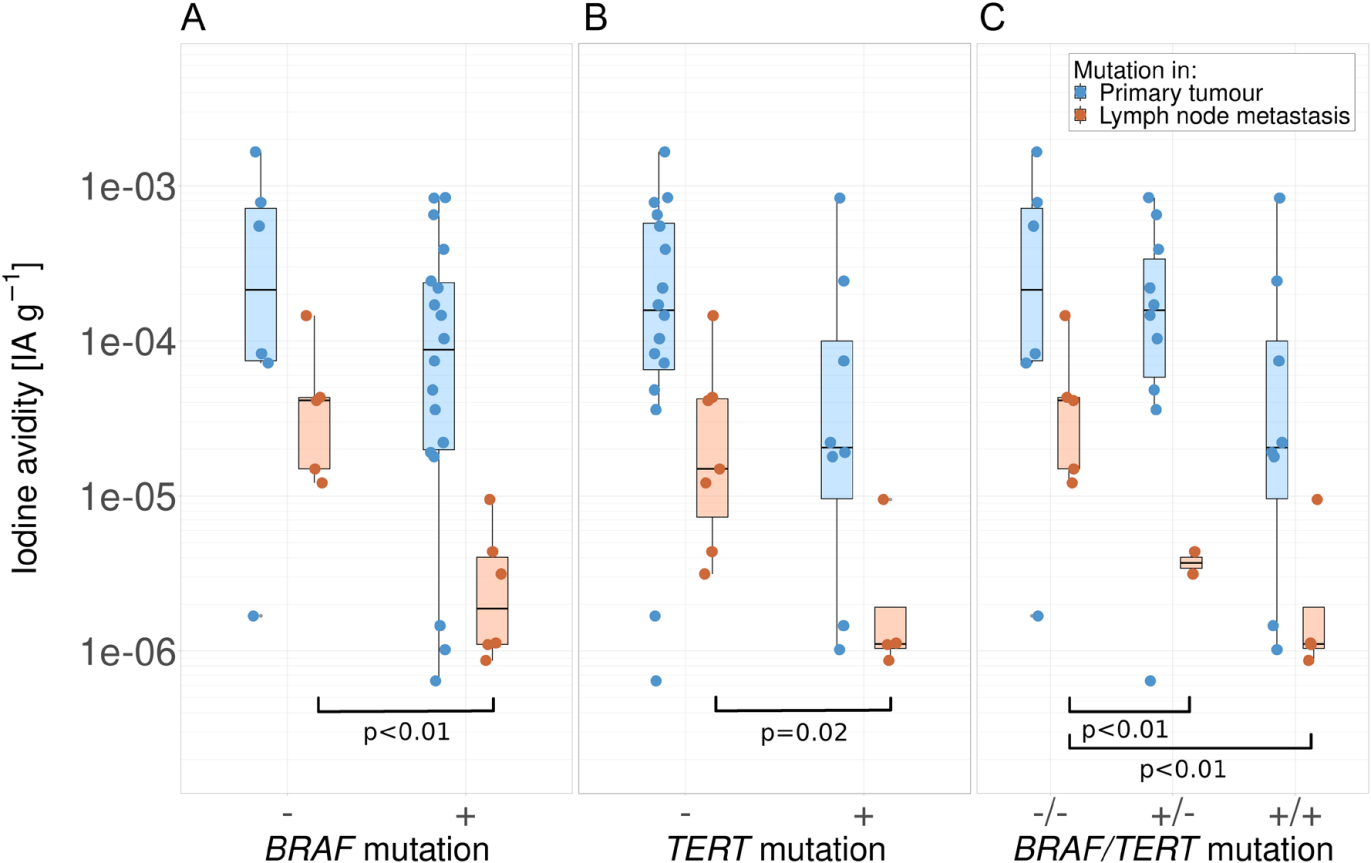
Iodine avidity (injected activity per gram tissue) in relation to *BRAF* V600E (A) and *TERT* promoter C228T (B) mutations. Co-occurrence of mutations is shown in (C). Significant differences in avidity were found between wildtype and mutated samples of lymph node metastases (orange markers). +, positive; −, negative.

*BRAF* V600E mutations were detected in 75% of primary tumours and in 55% of lymph node metastases. A statistically significant 18-fold lower avidity was observed in *BRAF*-mutated lymph node metastases (CI 3.9–87). The *TERT* promoter C228T mutation was found in 33% of primary tumours and in 36% of lymph node metastatic samples. *TERT* promoter mutations in lymph node metastases were significantly associated with lower iodine avidity, with a 10-fold lower avidity (CI 1.7–60). The results for iodine avidity in patients with combined *BRAF* and *TERT* promoter mutations showed a significantly lower avidity for lymph node metastases, with a 19-fold lower avidity for combined mutations (CI 3.4–110). All results for mutations are shown in Fig. 2. No significant difference in iodine avidity was observed for either mutation in primary tumour samples. A single *TERT* promoter C250T mutation was found in one sample, which also exhibited a C228T mutation. Mutational status in primary tumours and lymph node metastases agreed in five out of eight, *BRAF* mutations were discordant in the remaining samples. No mutations in *RAS* genes were observed in the *BRAF* mutated study population.

**Figure 3:**
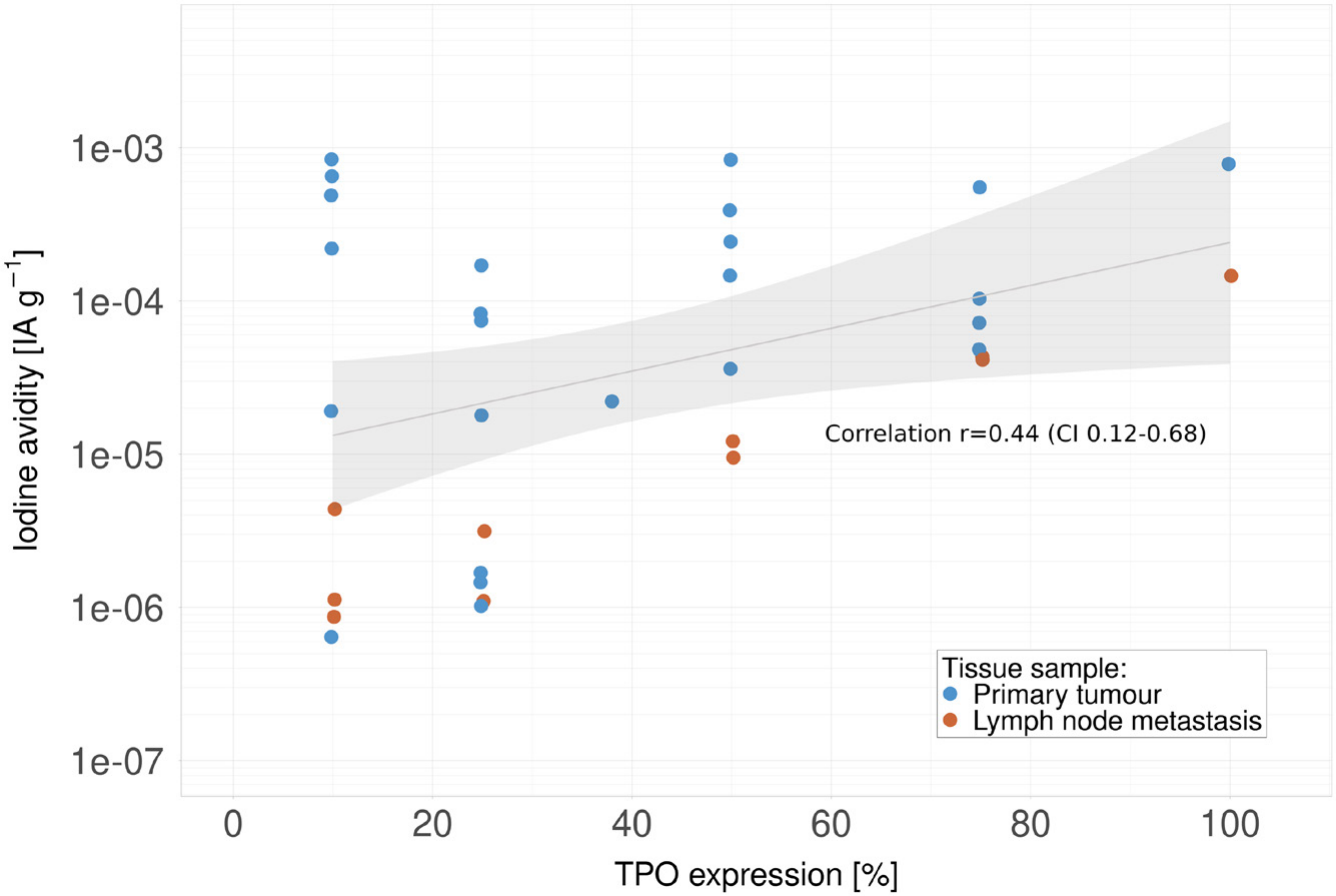
Iodine avidity (injected activity per gram tissue) in relation to the proportion of tumour cells expressing TPO in primary tumour and lymph node metastasis samples. A significant correlation between iodine avidity and TPO expression of *r* = 0.44 (CI 0.12–0.68) was observed. The log-linear fit is displayed along with confidence intervals (shaded area). TPO, thyroid peroxidase.

TPO expression had a considerable variance in the cohort and correlated with both iodine avidity (*r* = 0.44, CI 0.12–0.68), shown in Fig. 3, and cytoplasmic NIS expression (*r* = 0.42, CI 0.10–0.67). *TPO* expression was the only thyroid-related marker significantly associated with *BRAF* V600E (36 percentage points lower TPO expression, CI 13–58) and *TERT* promoter mutations (19 percentage points lower TPO expression, CI 3–35).

**Figure 4:**
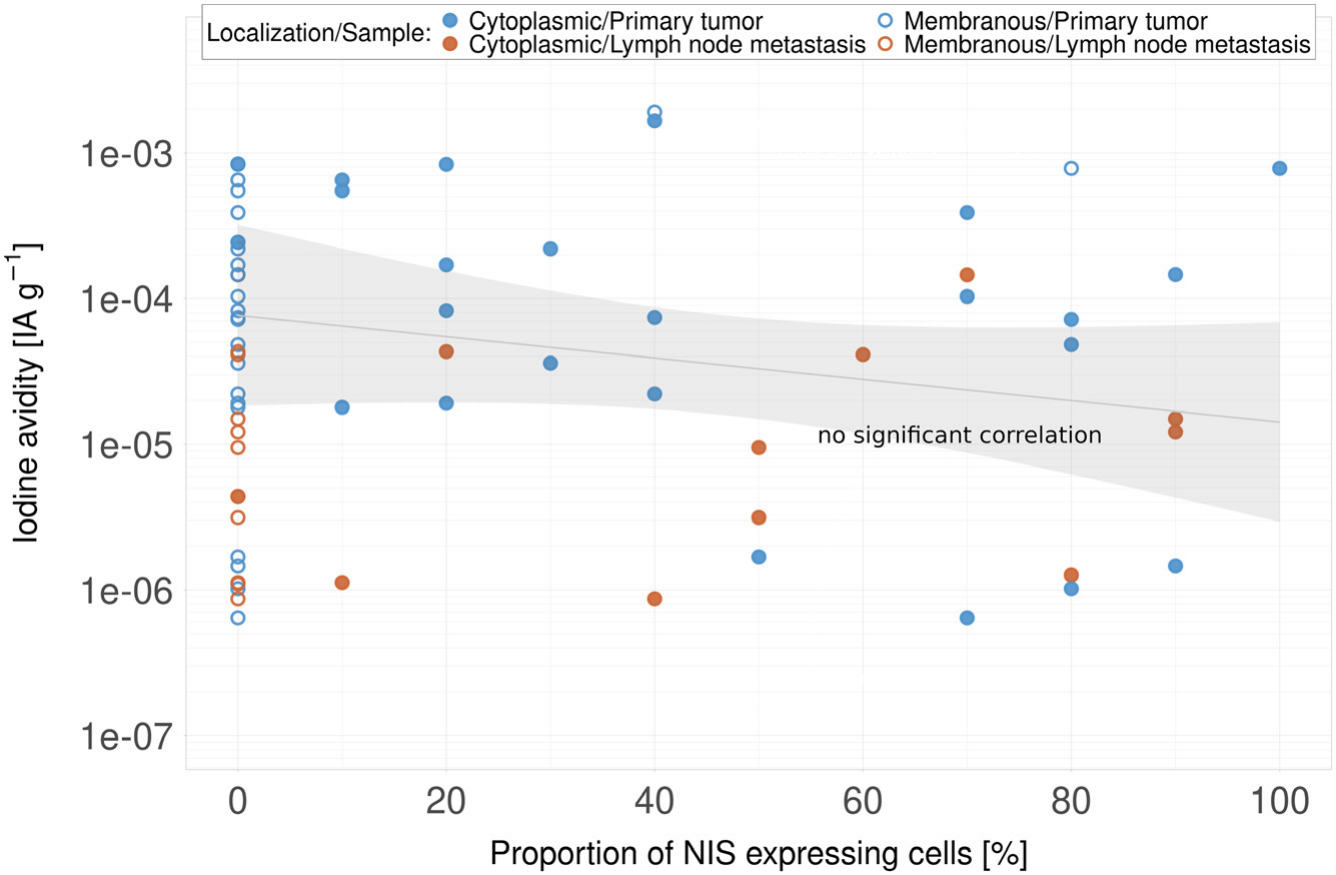
Relation between the proportion of cells in primary tumours and lymph node metastases with NIS expression as assessed by immunohistochemistry and iodine avidity (injected activity per gram tissue). Separate data points are shown for cytoplasmic and membranous localisation of NIS staining. No significant correlation was found between cytoplasmic NIS expression and iodine avidity. The log-linear fit is displayed for cytoplasmic staining, along with confidence intervals of the fit (shaded area).

NIS expression was found in some primary tumours and the majority of metastatic lesions, as shown in Fig. 4. The signal was predominantly cytosolic, with only two primary tumour samples demonstrating a clear-cut membranous signal. The samples with membranous NIS signals were *BRAF* and *TERT* promoter wildtype. The two samples with membranous NIS expression had a statistically significant 40-fold higher iodine avidity than those without membranous NIS expression (CI 9.1–180). No significant correlation was found between cytosolic NIS expression and iodine avidity (*r* = −0.23, CI −0.52 to 0.11). NIS expression was not found to be significantly different in patients with *BRAFV600E* or *TERT* promoter mutations.

TSHR expression was present to some degree in all samples, and pendrin was almost universally expressed (the only exceptions were two samples of PDTC). TSHR expression was moderately correlated with iodine avidity (*r* = 0.34, CI 0.01–0.61) while pendrin showed no correlation with iodine avidity (*r* = 0.10, CI −0.24 to 0.42).

### Multivariate regression

The multivariate regression showed that a model using expression of TPO and NIS, mutation status of the *TERT* promoter, high-risk histology (tall cell PTC, PDTC and DHGTC) and tumour tissue localisation (primary tumour/lymph node) performed very well in predicting iodine avidity. The parameters of the model are shown in Table 4. The model had an adjusted *R*^2^ value of 0.68 (*P* < 0.01), implying that as much as two-thirds of the variation in iodine avidity between patients may be predicted from surgical material. The model had low collinearity (all variance inflation factors below 1.6), residuals were normally distributed and no heteroskedasticity was found (Goldfeld-Quandt test *P* = 0.72). Removing most variables from the model caused a significant drop in adjusted *R*^2^, but the model was found to perform similarly well (adjusted *R*^2^ of 0.66) if *TERT* promoter mutation was replaced with thyroglobulin expression.

**Table 4:** Results of the linear multivariable regression for iodine avidity.

Parameter	Estimate	95% Confidence interval	*P*-value
	(fold-difference)		
Tissue localisation (primary tumour)	8.6	2.6–27	<0.01

High-risk histology (tall cell subtype PTC, PDTC, DHGTC)	0.27	0.09–0.87	0.03

TPO expression (per 10% of cells)	1.4	1.2–1.8	<0.01

Cytoplasmic NIS expression (per 10% of cells)	0.71	0.59–0.85	<0.01

Membranous NIS expression (per 10% of cells)	1.4	1.0–2.1	0.08

*TERT* promoter mutation (C228T)	0.36	0.12–1.1	0.06


The model had an adjusted *R* ^2^ of 0.68 and a *P*-value of <0.01. The model predicts that iodine avidity can be expected to be 40% higher for every 10% of cells that expressed TPO. Similarly, iodine avidity is modelled to be almost 4-fold lower if the tissue specimen was of high-risk histological type.

DHGTC, differentiated high-grade thyroid cancer; NIS, sodium–iodide symporter; PDTC, poorly differentiated thyroid cancer; PTC, papillary thyroid cancer; TPO, thyroid peroxidase.

## Discussion

This work includes, to the authors’ knowledge, the first published data on the link between quantitative radioiodine avidity in surgical specimens, mutations in *BRAF* and the *TERT* promoter and the expression of NIS, TPO, TSHR and pendrin. The unique study design enabled detailed quantification and modelling of iodine concentrations in the same tumour tissue that underwent histopathological examination.

The current work showed that several independent parameters coincide to explain why iodine avidity is found to be much lower in many thyroid cancers. Of major impact was the expression of TPO and NIS, which can be expected because of their central part in concentrating and storing iodine in thyroid cells. Thyroid cancer cells are known to have variable and generally lower expression of TPO, which may have prognostic implications (20, 21). The current work indeed found variable expression of TPO, but our results also suggest that TPO can serve as a marker for the degree of iodine avidity in tumour tissue. NIS expression has been studied in relation to iodine avidity in metastatic lesions previously, finding links with iodine uptake on post-therapeutic whole-body scintigraphy (22, 23, 24, 25). *NIS* mRNA levels have been linked to both *TSHR* mRNA and tumour marker *Thyroglobulin* mRNA levels (26, 27). Similarly to results from Tavares *et al.*, the current work found NIS located at the basolateral membrane only in tumours of *BRAF, TERT* promoter and *RAS* wildtype (28). Since many samples in our cohort had concentrated iodine far above blood concentration, some level of NIS must have been present in the tumour cell membranes to mediate the transport, despite us being unable to detect it on immunohistochemistry with our methodology. The use of methods such as immunohistoflourescence or proximity ligation assays with higher sensitivity might enable further analysis of the presence of membranous NIS. Another possible explanation could be an unknown anion transport mechanism that may contribute to the observed iodine accumulation in cells with no observable membranous NIS. To exclude poor antigenicity at the plasma membrane level, subsets of NIS-negative tumours were stained for E-cadherin and beta-catenin, all displaying strong and diffuse membranous staining – thereby suggesting that poor fixation is not a factor when determining NIS expression. Moreover, the risk of ‘stunning’ was considered when choosing the activity of radioiodine prior to surgery. The used activities were well below what has previously been shown to not affect NIS mRNA levels (Supplementary Material 2) (18).

The TSH receptor is known to vary in expression in thyroid cancer tissue, but to scarcely be absent, even in dedifferentiated tumours (29). TSHR expression was only found to be moderately correlated with reduced iodine avidity in this work and was not independent of other better-performing parameters in the prediction of iodine avidity. Pendrin was substantially expressed in most samples in our cohort, regardless of iodine avidity, which is in line with previous research (30).

Lymph node metastases with *BRAF* and *TERT* promoter mutations were found to exhibit lower iodine avidity, with differences higher than previously reported. Previous studies have shown that the *BRAF* V600E mutation, and the subsequent activation of the MAPK and PI3K/Akt/mTOR pathways, is linked to lower iodine avidity in metastases (31, 32, 33, 34). Similarly, *TERT* promoter mutations are associated with lower radioiodine uptake and worse patient outcome (35). Furthermore, the combination of *BRAF* and *TERT* promoter mutations may have a synergistic negative effect, with lower avidity and survival rates (6, 8, 9, 36, 37). The synergistic effect of both mutations was not observed in the current work. The results in the current work did show a similar trend as reported by Yang *et al.* and Meng *et al.* that calculated quantitative measures of tumour-to-background radioiodine uptake based on post-therapeutic scintigraphy (8, 9). Their two studies found a nine-fold and five-fold lower uptake in tumours harbouring *TERT* promoter mutations. Yang *et al.* also studied *BRAF* mutations and found a four-fold lower uptake in *BRAF*-mutated tumours. The differences in the current work were higher throughout, at 18-, 10- and 19-fold lower iodine avidity in lymph node metastases for mutations in *BRAF*, *TERT* promoter and their combination. This difference may be explained by a larger dynamic range or lack of background signal in the current method or by chance, since the previously reported values are encompassed by the reported CIs.

The multivariable modelling shows that a substantial amount, perhaps up to two-thirds, of the variation in iodine avidity between patients can be accounted for ahead of initial radioiodine treatment. This has the potential to improve on current clinical management, where standard amounts (1.1, 3.7 and 7.4 GBq) of radioiodine are given, mainly based on pTNM staging. While TNM is a classification intended to stratify according to aggregated risk, the knowledge of iodine avidity enables individualising treatment to the characteristics, and expected treatment benefit, of the individual patient.

One difference in the current work from the therapeutic setting is that no TSH stimulation was used. The patients were in a euthyroid state at the time of iodine-131 injection, which corresponds to lower TSH than in publications that studied post-therapeutic scintigraphies. Under strongly elevated serum TSH levels, such as after TSH stimulation in preparation for radioiodine treatment, iodine concentrations would probably have been higher, as TSH is known to mediate the transfer of NIS to the plasma membrane (38, 39). It is worth noting that previous research has used therapy-related imaging after TSH stimulation and compared it to samples acquired under euthyroid conditions. Since in this study, all material was collected under a euthyroid state, the results add coherence to the analysis by performing specimen collection and avidity assessment simultaneously.

One strength of the current study is the use of tumoural iodine concentration as a measure of iodine avidity, which enabled both precise quantification and molecular analyses to be performed on the same tissue. A limitation of the study is the relatively small number of participants, which may have hindered the detection of parameters with modest, but perhaps important, effects on iodine avidity. Further studies with larger patient series to confirm the predictive value of these parameters are therefore warranted.

In conclusion, we have shown that an extended immunohistochemical and molecular work-up can identify tumours with reduced iodine avidity in metastatic thyroid cancer using an *ex vivo* experimental design. The loss of avidity seems to be linked to the loss of TPO expression, alterations in NIS expression, the gain of high-risk histology and *TERT* promoter mutations. These results could enable adaptation to the expected radioiodine treatment effect and inform the choice of radioiodine activity.

## Supplementary Materials

Supplementary Material 1

Supplementary Material 2

Supplementary Material 3

## Declaration of interest

No potential conflicts of interest relevant to this article exist.

## Funding

The study was financially supported by grants from the Swedish Cancer Society and Medical Diagnostics Karolinska.

## Author contribution statement

JNN: Conceptualisation, data collection, analysis, methodology, software, visualisation, writing; JS: Conceptualisation, methodology, writing; VC: Data collection, methodology, writing; KJ: Data collection, methodology, writing; RS: Data collection, methodology, writing; CH: Conceptualisation, writing; CIL: Conceptualisation, methodology, writing; CCJ: Conceptualisation, data collection, analysis, methodology, visusalisation, writing.
