# Supplementary material for: Iodine avidity in papillary and poorly differentiated thyroid cancer is predicted by immunohistochemical and molecular work-up
Source: Eur Thyroid J. 2023 Jul 28;12(4):e230099. doi: 10.1530/ETJ-23-0099 (PMC10388652; doi:10.1530/ETJ-23-0099)
Supplement: Supplementary Material 1 [file supplementary_material_1.pdf]

Supplementary material 1 - Material and methods details

Sample and radioactivity handling

Patients were asked to adhere to an iodine-restricted diet for one week before the injection, with the aim of limiting variance in uptake due to dietary iodine load. No thyroid stimulating hormone (TSH) stimulation was given to patients prior to the radioactive iodine, as patients receiving potent exogeneous TSH stimulation before surgery could risk having an induced thyrotoxicosis and subsequent higher risk of associated surgical complications. While the study methodology in general attempted to mimic the therapeutic setting where possible, the risks of TSH stimulation was not clearly outweighed by the potential benefits, as having the healthy thyroid present would still limit any direct comparison to the therapeutic setting.

After surgery, representative pieces of tumour and lymph node metastases were dissected by an experienced surgical pathologist or a specialised pathology laboratory assistant. In the case of multifocal primary tumour growth, the largest lesion was dissected. When feasible, multiple samples were taken from both the primary tumour and initial lymph node metastases to calculate averages of iodine concentrations, to limit the impact of random sampling in heterogeneous tumours. The iodine concentrations in representative tissue samples were measured using a calibrated NaI(Tl)-scintillation well chamber. The radioactivity in tumour samples was quantified as normalised activity concentration (fraction of injected activity per gram of tissue: IA g^{-1}).

Any non-tumoural components in the measured sample were corrected for by weight subtraction. The concentration was also corrected for competing uptake in normal thyroid tissue, as some patients had dominant accumulation of iodine in normal tissue. Therefore, similarly to biodistribution studies in small animals where activity can extravasate in significant amounts, the measured uptake was divided with the fraction of available iodine (excluding that in normal tissue) to improve comparison between patients.

Normal thyroid tissue taken at grossing served as an internal control for each patient, where the thyroid uptake could confirm the reliability of the measurements. The median normal thyroid tissue uptake was 38% after 2 days (IQR 25-52%), corresponding roughly to what could be expected in a population adhering to a low-iodine diet. The results of these control measurements are shown below in Figure 1.

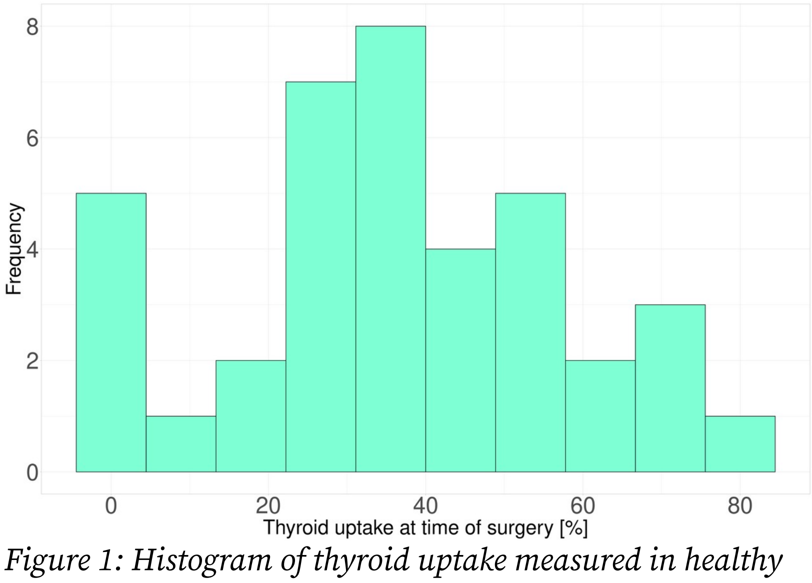

Figure 1: Histogram of thyroid uptake measured in healthy gland at surgery. Whole-gland uptake was estimated by multiplying %IA/g (determined in representative samples) with whole-gland mass estimated at pathology grossing (total mass minus estimated primary tumour mass).

Molecular analyses

DNA was extracted from formalin-fixed paraffin-embedded (FFPE) tissue sections. Tissue blocks were cut and 3-6 sections 10 um thick were used (number of sections depending on the tumor size). DNA was extracted with the FFPE DNA extraction kit using Maxwell RSC DNA FFPE KIT (AS1450) (Promega, Madison, Wisconsin, USA). Sections were then incubated in 180 ul of incubation buffer and 20 ul Protienase K for two hours. After that, 400 ul of lysis buffer was added, and DNA was extracted using the Maxwell 16 robot (AS2000) (Promega, Madison, Wisconsin, USA). The quantity and quality of the DNA was measured by using Nanodrop (ThermoFisher Scientific, Waltam, Massachusetts, USA).

TERT promoter mutational analysis of DNA from FFPE samples was performed using ddPCR (QX200 Droplet Digital PCR system; Bio-Rad Laboratories, Hercules, CA, USA) together with validated ddPCR mutation assays for detection of mutations corresponding to upstream positions -124 (C228T) and -146 (C250T) of the TERT promoter region. The DNA input was 25 ng per reaction. Mutation-positive and mutation-negative references were male genomic DNA (Horizon Discovery, Cambridge, UK).

All samples were screened for BRAF V600E through PCR analysis on the Cobas z480 analyzer for automated amplification and detection using the Cobas 4800 BRAF V600 Mutation Test (Roche Diagnostics GmbH, Germany). All positive mutational findings were verified through additional real-time PCR screening for presence of hot spot mutations in KRAS (codons 12 and 13), NRAS (codon 61), and HRAS (codons 12, 13, and 61) with the EntroGen Thyroid Cancer Mutation Analysis Panel Kit (EntroGen, Woodland Hills, CA) using the protocol provided by the manufacturer.

Immunohistochemistry

Formalin-fixed paraffin-embedded (FFPE) tissues from all thyroid samples were sectioned in 4 µm thick sections followed by deparaffinization in xilene, and rehydration in ethanol. All sections were subjected to antigen retrieval in tris-EDTA buffer, pH 8.0 (Sigma, E-1161) at 95 °C for 15 minutes. Slides were incubated at room temperature with hydrogen peroxide, followed by 15 minutes blocking step using Background Sniper (BS966; Biocare Medical).

Primary antibodies anti-TSH-R (1:2000; ab218108, Abcam), anti-TPO (1:4000; ab133322; Abcam), anti-SLC26A4 (1:2000; PA542060; Life Technologies), and anti-NIS (1:1000; ab242007; Abcam) were diluted in Renoir Red diluent (PD904; Biocare Medical) and incubated at 4°C overnight. MACH-1 Universal HRP-Polymer Detection kit (M1U539; Biocare Medical) was used for the detection step according to the manufacturing protocol. Counterstaining with hematoxylin as well as dehydration in ethanol and xylene was performed. De-identified cases of Graves' disease patients were used as positive controls for each marker.

The mutation-specific V600E BRAF staining procedure was performed in a clinically accredited pathology laboratory setting using an anti-BRAF V600E (VE1) mouse monoclonal primary antibody and standardized methodology via a Ventana automated staining system (Roche, Basel, Switzerland). The staining was performed and scored in a clinical routine setting and was included in the analysis for validation purposes.

The plasma membrane proteins E-cadherin and beta-catenin were included to demonstrate intact antigenicity for membrane markers in subsets of NIS negative cases. Both antibodies are routinely used in our daily pathology practice at the Karolinska University Hospital, and were thus stained

using automated Ventana methodology. E-cadherin (clone 36) is a mouse monoclonal antibody used with a ready-to-use dilution with antigen retrieval using CC1 for 32 mins (Roche, Basel, Switzerland) and incubation time of 4 mins. Beta-catenin (clone 14) is a mouse monoclonal antibody used with a ready-to-use dilution with antigen retrieval using CC1 for 64 mins (Roche, Basel, Switzerland) and incubation time of 52 mins. External controls consisted on various de-identified tissues used in clinical routine (data not shown).
