# Supplementary material for: Iodine avidity in papillary and poorly differentiated thyroid cancer is predicted by immunohistochemical and molecular work-up
Source: Eur Thyroid J. 2023 Jul 28;12(4):e230099. doi: 10.1530/ETJ-23-0099 (PMC10388652; doi:10.1530/ETJ-23-0099)
Supplement: Supplementary Material 2 [file supplementary_material_2.pdf]

Supplementary material 2 - Absorbed dose to tumour tissue

<u>Assumptions</u>

Injection 10 MBq I-131 (max activity given)
Uptake 0.2%/g in tumour tissue (highest measured tumour uptake in the cohort)
Nothing leaves the tissue during the 48 h until surgery (conservative)
All beta energy stays in tumour tissue (slightly conservative)
192 keV deposited per decay

Results in:
0.1 Gy to tumour tissue
Lundh et al. (doi:10.2967/jnumed.108.061150) showed that at 0.5 Gy given with I-131 to normal thyroid cells in culture, no significant reduction in NIS mRNA expression could be observed at 1 d. An effect was seen at 5 d, however. Considering that 0.1 Gy was our "worst case", no stunning was expected to interfere with either the uptake (which in any case had already happened), nor NIS expression at surgery.

TABLE 2. Changes of NIS mRNA Expression in Thyroid Cells Irradiated with ^{123}I, ^{131}I, ^{99m}Tc, or ^{211}At to Absorbed Dose of 0.5 Gy During 6 Hours, on Days 1 and 5 After Irradiation

	NIS mRNA downregulation (% of control)	
Radionuclide	1 d	5 d
^{131}I	NS	80
^{123}I	55	NS
^{99m}Tc	NS	34
^{211}At	26	61

qRT-PCR data are presented as percentage downregulation, compared with matched nonirradiated controls. NS indicates no statistically significant difference from controls, $P < 0.001$.

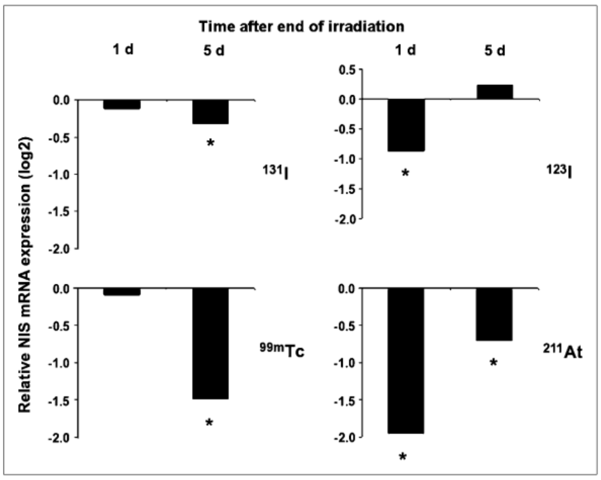

FIGURE 3. Changes of NIS mRNA expression in thyroid cells irradiated with ^{123}I, ^{131}I, ^{99m}Tc, or ^{211}At at same dose (0.5 Gy) and exposure times as shown in Figure 2 (representing data from parallel cultures in same experiments) on days 1 and 5 after irradiation. qRT-PCR data are presented as log2 expression levels compared with those of matched nonirradiated controls ($n = 3$). *Statistically significant difference from controls, $P < 0.001$.
