# Supplementary material for: Iodine avidity in papillary and poorly differentiated thyroid cancer is predicted by immunohistochemical and molecular work-up
Source: Eur Thyroid J. 2023 Jul 28;12(4):e230099. doi: 10.1530/ETJ-23-0099 (PMC10388652; doi:10.1530/ETJ-23-0099)
Supplement: Supplementary Material 3 [file supplementary_material_3.pdf]

Supplementary material 3 - Immunohistochemistry controls

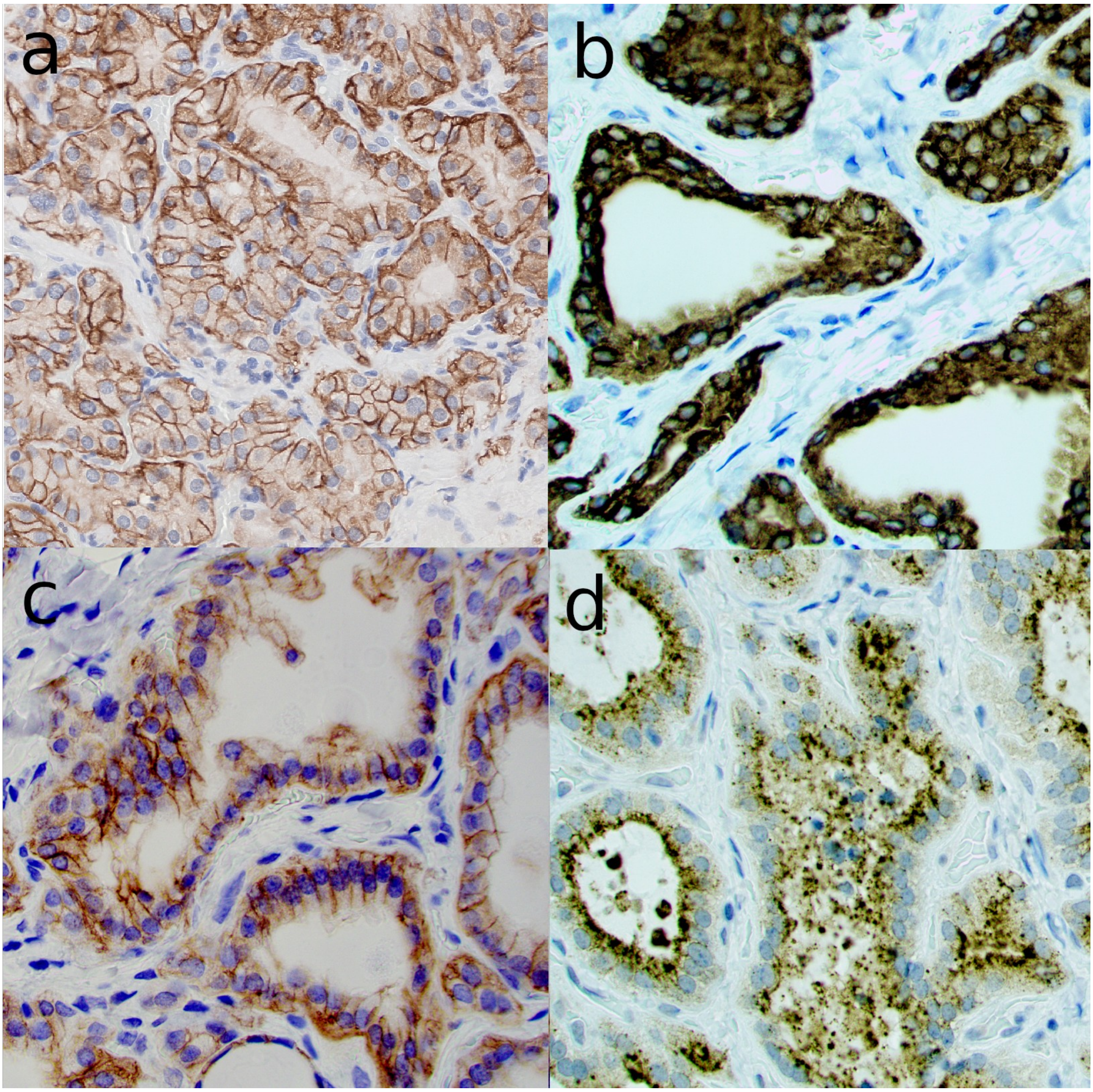

Figure 1: Immunohistochemistry in four samples of Graves' Disease. NIS (a), TPO (b), TSHR (c) and pendrin (d) all showed intense signal as expected.

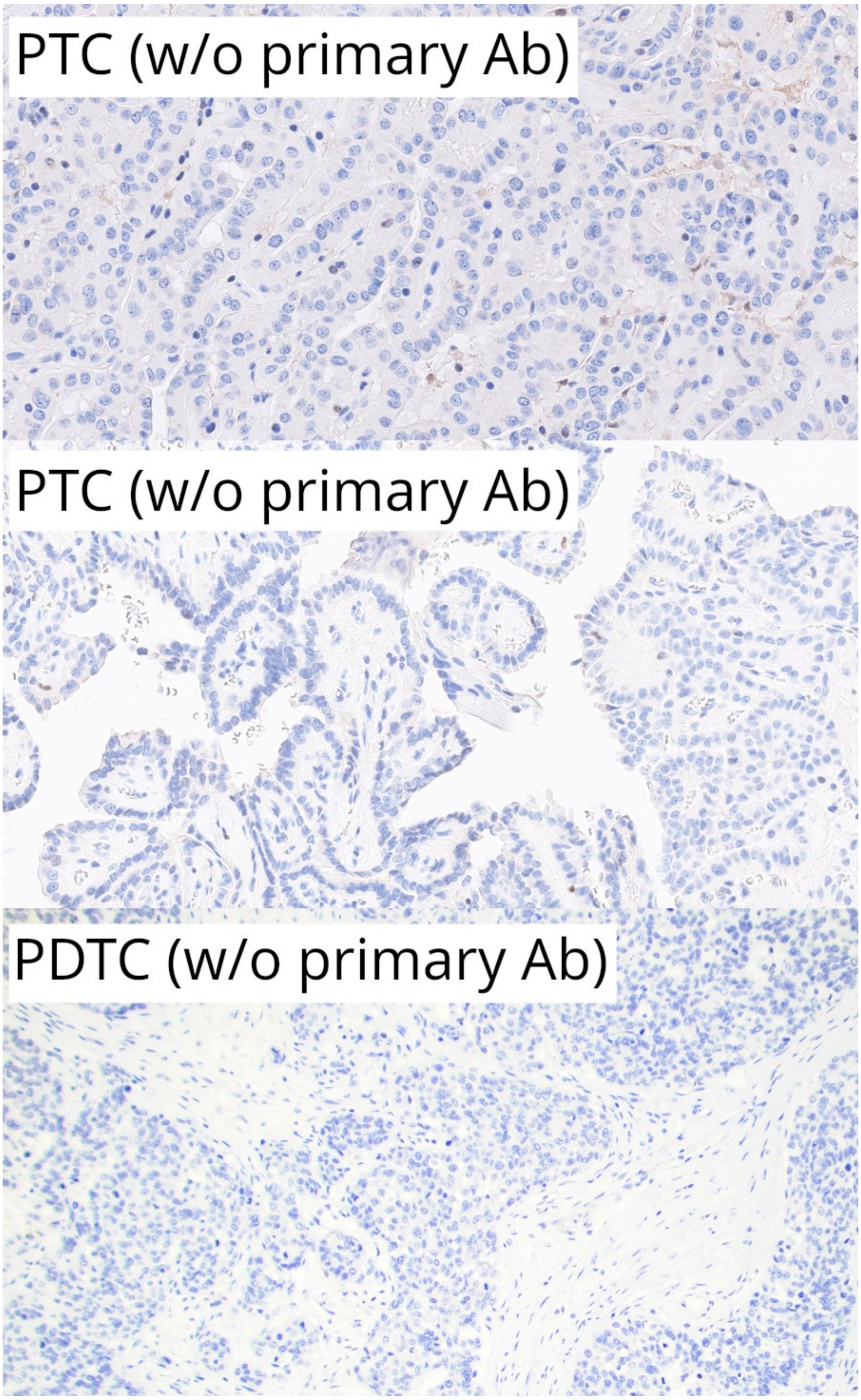

Figure 2: Immunohistochemistry in three of the studied tumour samples, omitting the primary antibody to confirm that no unspecific binding occurred.

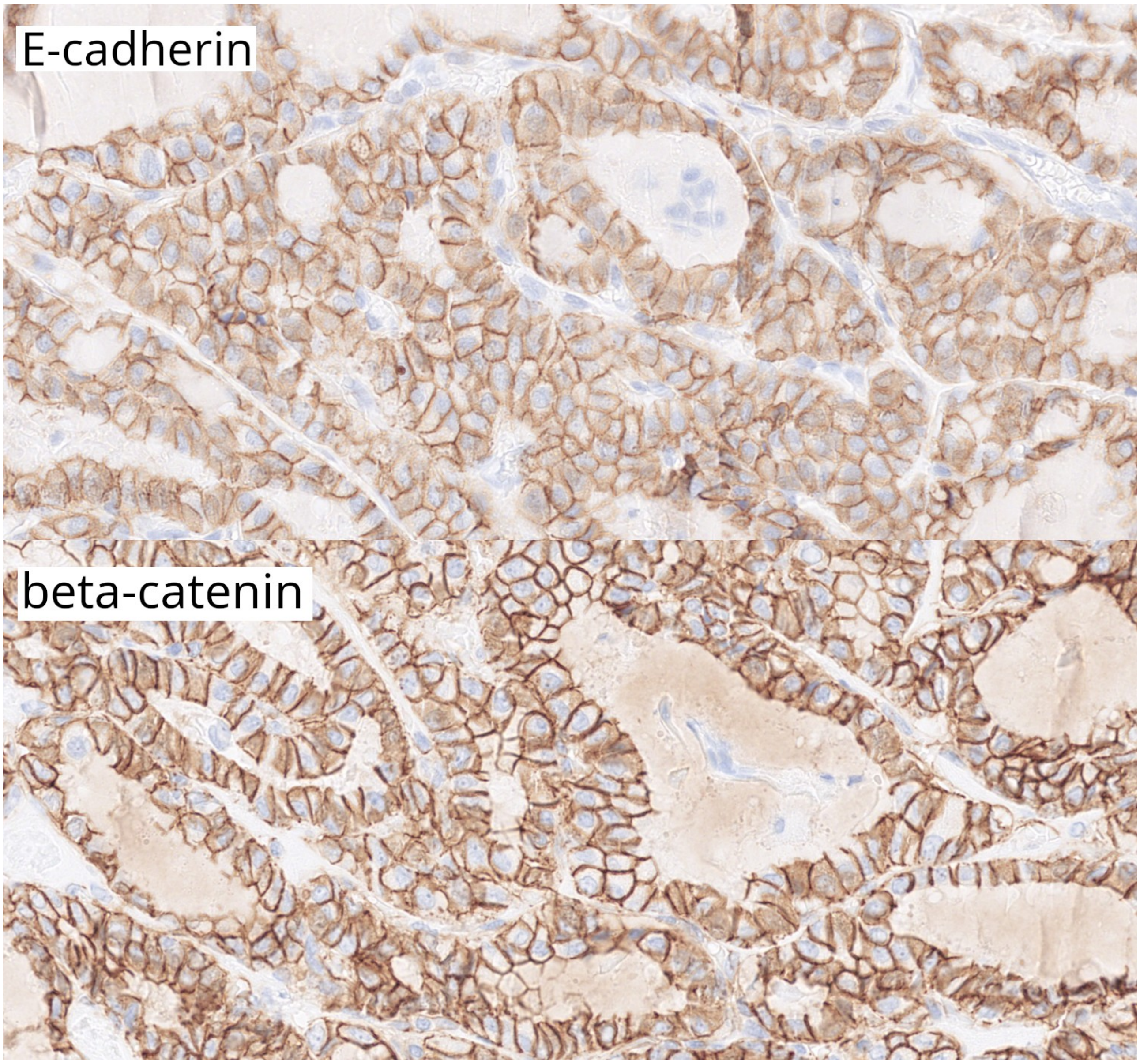

Figure 3: Immunohistochemistry with cellular plasma membrane markers E-cadherin and beta-catenin.
